# Guide device to assist in performing arthroscopic surgery of the temporomandibular joint—a preliminary study

**DOI:** 10.22514/jofph.2025.012

**Published:** 2025-03-12

**Authors:** Waseem Abboud, Shoshana Reiter, Pessia Friedman-Rubin, Dror Shamir, Oren Peleg

**Affiliations:** ^1^Department of Oral and Maxillofacial Surgery, Goldschleger School of Dental Medicine, Faculty of Medicine, Tel Aviv University, 6997801 Tel Aviv, Israel; ^2^Unit of Oral and Maxillofacial Surgery Bnei Zion Medical Center, Affiliated to Technion Faculty of Medicine, 3339419 Haifa, Israel; ^3^Department of Neurology, Sheba Tel-Hashomer Medical Center, Institute of Movement Disorders, Affiliated to Faculty of Medicine, Tel Aviv University, 5262000 Tel Aviv, Israel; ^4^Department of Oral Pathology, Oral Medicine, and Maxillofacial Imaging, Goldschleger School of Dental Medicine, Faculty of Medicine, Tel Aviv University, 6697801 Tel Aviv, Israel; ^5^Department of Oral Rehabilitation, Goldschleger School of Dental Medicine, Faculty of Medicine, Tel Aviv University, 6697801 Tel Aviv, Israel; ^6^Department of Otolaryngology Head and Neck Surgery and Maxillofacial Surgery, Sourasky Medical Center, Affiliated to Faculty of Medicine, Tel Aviv University, 6423910 Tel Aviv, Israel

**Keywords:** Arthroscopy, Arthroscopic surgery, Temporomandibular joint, TMJ, Internal derangement, Guide device

## Abstract

**Background**: Arthroscopic surgery of the temporomandibular joint (TMJ) requires inserting an arthroscope and a working cannula into the joint cavity. Working cannula introduction and positioning require high levels of expertise. **Methods**: A randomized controlled trial was performed on patients with closed lock of the TMJ who underwent arthroscopic lysis and lavage. A total of 15 subjects participated in this study, with 6 in the study group using the Locator-Positioner guide device (LOPO) and 9 in the control group using triangulation. The main outcomes included: (1) Number of attempts necessary for successful cannula insertion. (2) The time between arthroscope insertion and the appearance of the working cannula on the monitor, and (3) Overall surgery duration. **Results**: A successful cannula insertion took an average of 2.1 attempts in the study group compared with 3 attempts in the control group (*p* = 0.045). Study group arthroscope insertion to monitor appearance of cannula took 2.3 minutes, whereas control group took 4 minutes (*p* = 0.039). A total of 14 minutes was spent on surgery in the study group compared to 16.5 minutes in the control group (*p* = 0.009). **Conclusions**: LOPO device improved both the insertion of the working cannula into the TMJ and its positioning relative to the arthroscope throughout surgery. It reduced insertion attempts and shortened the surgery duration. **Clinical Trial Registration**: the study was registered at clinicaltrials.gov, identifier: NCT 06520917.

## 1. Introduction

Temporomandibular joint (TMJ) arthroscopy allows direct visualization and access to the joint, enabling surgeons to diagnose and perform a wide range of procedures. A diagnostic arthroscopy involves inserting a cannula into the joint cavity to accommodate an arthroscope. In contrast, arthroscopic surgery requires insertion of at least two portals into the joint cavity; an arthroscopic cannula and a working cannula, where the latter is intended to incorporate instruments into the joint space [1, 2, 3]. In most cases, the arthroscopic cannula is inserted via a lateral approach into the posterior recess of the superior joint space. As the glenoid fossa is easily palpated and the target point is just deep into the skin and joint capsule, this is an easy puncture [4]. The second or working cannula is inserted into the anterior recess of the superior joint space. A precise and accurate skin puncture site, insertion angle and insertion depth are necessary to ensure the second or working, cannula is readily functional and visible to the arthroscope. This alignment allows the cannula tip to meet and be seen with an arthroscope [5, 6]. In the second puncture, the site and technique are dictated by the anatomy and volume of the anterior recess of the superior joint space, which varies greatly between individuals and joint conditions. The target point on the skin is also the juncture between the anterior aspect of the anterior slope of the articular eminence and the continuation of the zygoma, which is usually not palpable. To overcome these difficulties, triangulation was adapted for TMJ by pioneer arthroscopists such as K Murakami and J P Mc Cain [7, 8]. This facilitates easier and more predictable entry of the second cannula into the joint cavity, which allows access to different areas in the joint while being fully visualized by the arthroscope.

Adherence to triangulation is essential to successful second or working cannula placement. This technique, however, requires a great deal of practice. Furthermore, maintaining the optimal spatial alignment of the working cannula relative to the arthroscope throughout surgery requires additional practice, experience and synchronized collaboration between surgeon and assistant [9, 10].

During arthroscopic surgery, precision in entering the TMJ and accuracy in aligning the arthroscopic cannula with the working cannula are key to minimizing iatrogenic damage. Multiple joint punctures due to repeated attempts to enter the joint cavity or scuffing of intraarticular surfaces due to re-alignment of the cannulas increase joint trauma risk. This exacerbates postoperative pain and edema, which is particularly problematic for patients suffering from TMJ-related pain. Since they must engage in immediate postoperative mobilization and full-range physiotherapy to prevent joint stiffness and promote functional recovery, any additional discomfort or inflammation caused by surgical inaccuracies can severely impact rehabilitation [11, 12]. The accuracy of cannula placement is therefore of paramount importance. By minimizing postoperative discomfort and facilitating early functional recovery, these measures not only protect the delicate intraarticular structures but also optimize patient outcomes.

A Locator-Positioner guide device (LOPO) was developed to address these challenges by the main author to enable surgeons to insert a working cannula and maintain it in three-dimensional position with respect to the arthroscope throughout surgery, while allowing both cannulas to move freely [13, 14]. This method helps minimize intraarticular scuffing and surgical risks by allowing easy and precise insertion and positioning of the working cannula. This study was conducted to examine the accuracy, safety and efficiency of the LOPO guide device.

## 2. Materials and methods

Two parallelograms form the two arms of the LOPO guide device. A connector holds both arms and permits movement by changing the angle between them (Fig. 1). The arthroscope is attached to one arm of the device, while the working cannula is to the other. After the arthroscope is introduced into the joint cavity, the guide device is mounted on the arthroscope base via one arm. The other arm receives the working cannula, and owing to the double parallelogram design, it directs it to the ideal puncture site, insertion vector and depth into the joint cavity, where it meets the tip of the arthroscope. The connector between the two arms of the device enables changing the angle between them while maintaining the relative position of the tip of the cannula to the tip of the arthroscope (Fig. 1).

**Fig. 1.** LOPO guide device attached to the arthroscope and working cannula. LOPO guide device appears in grey and the arthroscope and working cannulas appear in red. The LOPO consists of two connected parallelogram-shaped arms; One arm attaches to the arthroscope after it is inserted into the joint cavity while the other is utilized to insert the working cannula, aiming it to the tip of the arthroscope inside the joint space. By adjusting the angle between the two arms of the LOPO, the arthroscope and working cannula can be aligned differently while remaining close to one another. Due to elastic pads attaching the cannulas to the LOPO device, micromovements (dashed red lines) are still possible in the fixed position after locking the connector of the LOPO.

The connector can also be locked, thereby fixing the angle between the two arms. Even in this locked position, the arthroscope and working cannula still maintain micromovements thanks to elastic pads attaching the cannulas to the arms. These flexible holders allow micromovements of the cannulas without changing the angle between the two arms.

In surgery, the surgeon holds the LOPO device with its two cannulas in one hand. Through the working cannula, the other hand is free to perform surgical instrumentation (Fig. 2). Due to the double parallelogram design, all this is accomplished while maintaining the ideal position of the working cannula relative to the arthroscope. The guide device can be easily detached from the arthroscopic cannula and working cannula. This can be done at any point during surgery without removing or altering the position of the cannulas, allowing them to remain securely within the joint. It is also possible to reattach the device to the cannula if necessary later in the procedure.

**Fig. 2.** Clinical pictures of the LOPO device. Demonstration of the device in real surgery at acute, straight and obtuse angles. The optimal spatial alignment of arthroscope tip relative to the working cannula is maintained through this full range of motion.

Approval for conducting an experiment with human subjects using the LOPO device was granted by the Institutional Review Board of Sheba Medical Center (4657-17-SMC) and the National Ministry of Health (MOH_2018-08-27_003619). The study was conducted at Sheba Medical Center. The study included 15 consecutive patients who underwent arthroscopic lysis and lavage for TMJ closed lock after unsuccessful conservative treatment. We provided verbal explanations as well as written forms to all patients and their family physician regarding the guide device. Participants signed informed consent forms and agreed to participate. A single arthroscopic procedure of the TMJ is routinely scheduled as part of a group of 2 to 3 procedures in the authors’ department due to technical issues. Patients were randomly assigned to the LOPO device based on the time they were admitted to the department; the first to arrive underwent an arthroscopy using LOPO guide device, while the other one or two patients on the same operation day were treated with the classic triangulation technique. Thus, six subjects were included in the experiment group and nine constituted the control group.

All arthroscopies were performed by the same surgeon under general anesthesia with nasotracheal intubation. The surgeon was an experienced TMJ arthroscopist and familiar with LOPO device and practiced using it on anatomical models (but not cadavers) before the clinical trial. A 2.4 mm 30° arthroscope with 84 mm cannula length (CE0123, HOPKINS II 28300 BA, Storz, Tuttlingen, BW, Germany) was used in all cases and the prototype of the LOPO device was manufactured based on the size and design of this instrument set. After inserting the arthroscope into the posterior recess of the superior joint space, the surgeon needed to insert the working cannula into the anterior recess of the superior joint space. For the control group, triangulation was used to insert the working cannula. Arthroscope insertion depth was measured on the skin. Nearly the same distance away from this point and parallel to the arthroscope on the skin was the second puncture site. The second cannula was then inserted perpendicularly to the skin, ensuring the triangulation principle was maintained [7, 8]. For the study group, the working cannula was inserted as follows: the LOPO device was mounted on arthroscope base after it was inserted into the posterior recess of the superior joint space. As directed and guided by the arm of the device, the working cannula was inserted into the joint cavity using the other arm of the LOPO device. The surgical procedure was arthroscopic lysis and lavage. Following the insertion of the working cannula, a diagnostic sweep was performed, followed by the mobilization of the disc using a blunt obturator. The lateral capsule and anterior attachments at the disc-capsule interface were stretched by the same blunt obturator. Finally, a subsynovial injection of steroids was performed under direct visualization.

The primary outcome variables were:

1. Number of attempts to successfully insert the working cannula into the joint cavity. After puncturing the skin and advancing to the joint, any partial or complete withdrawal of the cannula followed by a change of vector or depth was considered an additional attempt. The working cannula was observed on the monitor to confirm successful insertion.

2. Duration between the arthroscope’s insertion into the joint cavity and the initial appearance of the working cannula on the monitor.

3. Overall surgery duration, starting with insertion of the arthroscope into the joint cavity and ending with the removal of both the arthroscope and working cannula outside the joint cavity.

As a secondary outcome variable, the interincisal distance was measured at 3 months postoperatively to determine the maximal mouth opening after surgery. Additionally, adverse events were documented.

Statistical analysis: Data was summarized with descriptive statistics by groups (study versus control). Continuous variables were presented as mean and categorical variables by number (%). The primary outcome variables were compared using independent Welch two sample *t*-tests to check for possible confounders and significant differences. The difference in means, confidence interval and *p* value was described. Paired *T* test was used to compare the secondary outcome variable before and after arthroscopy for each group. *p * < 0.05 indicates statistically significant differences. Data analysis was performed using R Statistical Software (v4.1.2; R Core Team 2021).

The LOPO device is Patent No. US 11,517,348 B2. The main author is the inventor of the device and the assignee is Sheba Tel Hashomer Medical Research Services Ltd. A prototype of the device was manufactured by Pollak Steinberg Engineers, Tel Aviv, Israel and was made of stainless steel 303. LOPO has not yet been manufactured to be distributed on the market.

## 3. Results

Fifteen consecutive subjects were included, six in the study group (LOPO device) and nine in the control group (triangulation technique). Table 1 lists the characteristics of the subjects. Age, gender, duration of joint lock and baseline maximal mouth opening were not statistically different between the study and control groups.

**Table 1.** Characteristics of the study and control groups.

	Study, N = 6^ǂ^	Control, N = 9^ǂ^	*p*-value^ˠ^
Age (yr)	37.7 (13.2)	27.6 (12.1)	0.2
Gender (female)	5 (83%)	8 (89%)	0.95
Laterality (right)	4 (67%)	6 (67%)	0.98
Duration of joint lock	7.5 mon (4.59)	7.0 mon (3.46)	0.8
Maximal mouth opening before surgery	28.5 mm (4.04)	27.4 mm (2.70)	0.9
Maximal mouth opening after surgery	41.8 mm (5.78)	41.5 mm (5.01)	0.9

*^ǂ^Mean (SD); ^ˠ^Welch Two Sample t-test; SD: standard deviation.*

Primary outcome variables:

Study group averaged 2.1 attempts to successfully insert the working cannula into the joint cavity, while control group averaged 3 attempts (*p* = 0.045) (Table 2).

**Table 2.** Primary outcome parameters.

	Study, N = 6^ǂ^	Control, N = 9^ǂ^	Difference^ˠ^	95% CI^ˠ,^^ѱ^	*p*-value^ˠ^
Number of attempts for successful insertion (SD)	2.1 (1.17)	3.0 (1.01)	0.9	−0.03, 2.6	0.045
Time from cannula insertion to screen appearance in minutes (SD)	2.3 (1.21)	4.0 (1.58)	1.7	0.10, 3.2	0.039
Total duration of surgery in minutes (SD)	14.0 (1.26)	16.5 (1.27)	2.5	0.64, 3.6	0.009

*^ǂ^Mean (SD); ^ˠ^Welch Two Sample t-test; ^ѱ^CI: Confidence Interval; SD: standard deviation.*

2.3 minutes elapsed between the arthroscope being inserted into the joint cavity and the working cannula appearing on the monitor in the study group and 4 minutes in the control group. A significant difference was found (*p* = 0.039) (Table 2).

In the study group, the total surgery duration, from placement of the arthroscope to removing the cannulas, averaged 14 minutes, whereas in the control group, it averaged 16.5 minutes. A significant difference was found (*p* = 0.009).

Secondary outcome variable:

Maximal mouth opening improved significantly for both groups. The mouth opening increased from a mean of 28.5 mm to 41.8 mm in the study group (*p * < 0.0001), and from 27.4 mm to 41.5 mm in the control group (*p * < 0.0001). The degree of improvement between both groups did not differ significantly (*p *> 0.9).

Adverse events: injuries to the ear, branches of the facial nerve, or skin were not observed in any of the patients.

## 4. Discussion

This study evaluated a Locator-Positioner guide device (LOPO) designed to help the surgeon accurately insert the working cannula into the joint space and keep it positioned relative to the tip of the arthroscope throughout the procedure. Both goals were achieved with accuracy and effectiveness. With the LOPO device, the arthroscope and working cannula could be moved freely. A significant reduction in the number of attempts and time spent until the working cannula was successfully inserted into the joint space was achieved using the LOPO device compared to triangulation. In addition, overall surgical time was significantly shorter.

In spite of the fact that 30% fewer insertion punctures and operative time may not seem dramatic, the actual benefit of LOPO is a decrease in scuffing of intra-articular surfaces and a decrease in surgical morbidity. The present study is not able to objectively quantify this outcome. It is well known that with each failed attempt to insert the working cannula properly into the joint cavity or with every additional maneuver to re-position the working cannula into the arthroscope’s field of view after getting “lost” in the joint space, some damage can occur to the joint surfaces and surrounding structures [15, 16, 17]. Consequently, these injuries lead to postoperative edema and pain, limiting patients’ ability to participate in immediate mobilizations and physical therapy exercises, both of which are critical for the procedure’s success [11, 12].

Surgeons participating in this study had several years of experience performing arthroscopic surgery by triangulation. LOPO’s advantages may be more evident if the surgeons are novice arthroscopists with less experience with triangulation. Correct positioning of the second or working cannula relative to the arthroscope by the triangulation technique has a long learning curve [6, 9, 10, 18, 19, 20, 21]. This surgical expertise can be developed using cadavers and other learning methods through numerous courses and training programs. Anatomical models and simulators have also been developed for the purpose of training surgeons [22, 23, 24, 25, 26, 27].

Few authors advocated the use of single-cannula arthroscopy (similar to salivary gland arthroscopy) to overcome the difficulties of triangulation. Due to its limited scope, this did not gain popularity in the maxillofacial community, and two-port arthroscopy is now considered absolutely essential for arthroscopic surgery [28, 29, 30, 31].

Triangulation still has a major intraoperative limitation even after mastery. Both the arthroscopic and working cannulas are held by the surgeon with both hands, while the assistant performs intra-articular instrumentation through the working cannula. Alternatively, if the surgeon operates the arthroscope with one hand while inserting intra-articular instrumentation through the working cannula with the other, the assistant must hold the working cannula and ensure its alignment with the arthroscope, requiring sensitivity and synchronization between the two operators [32, 33, 34, 35]. By connecting both cannulas into one unit, the LOPO device overcomes this major limitation by allowing the surgeon to hold it while performing intra-articular instrumentation with the other hand. This is a significant upgrade in the surgical setting, enabling a single surgeon to perform the operation alone.

## 5. Conclusions

The LOPO guide device demonstrated effectiveness, safety and accuracy for TMJ arthroscopic surgery. An improved ability to position the working cannula within the joint space, reduced operation time, and a decrease in scuffing of intra-articular surfaces to a lower surgical morbidity rate and an easier post-surgical recovery. The surgeon can also reliably position both cannulas in an ideal spatial orientation using only one hand, allowing the other to work on intraoperative instrumentation.

However, there are some limitations to be aware of. Experienced surgeons may find the device cumbersome, potentially disrupting established techniques. Novice surgeons on the other hand may over-rely on the device, limiting their motivation to master traditional triangulation techniques. Furthermore, this additional device adds to the cost.

A future randomized controlled trial with novice surgeons and a larger patient group is recommended to further validate the efficacy and safety of the LOPO device. For future trials to provide more comprehensive and robust conclusions regarding the device’s effectiveness across a wide range of arthroscopic designs and sizes, developing guide devices compatible with different arthroscope sizes and manufacturers will be essential.

## References

[1] McCain JP, Montero J, Ahn DY, Hakim MA. Arhroscopy and arthrocentesis of the temporomandibular joint. In Miloro M, Ghali GE, Larsen P, Waite P (eds.) Peterson’s principles of oral and maxillofacial surgery (pp. 1569–1624). 4th edn. Springer: Cham, Switzerland. 2022.

[2] Liu X, Zheng J, Cai X, Abdelrehem A, Yang C. Techniques of Yang’s arthroscopic discopexy for temporomandibular joint rotational anterior disc displacement. International Journal of Oral and Maxillofacial Surgery. 2019; 48: 769–778. 10.1016/j.ijom.2018.12.003 30594477

[3] Abboud W, Yahalom R, Givol N. Treatment of intermittent locking of the jaw in Wilkes Stage II derangement by arthroscopic lysis and lavage. Journal of Oral and Maxillofacial Surgery. 2015; 73: 1466–1472. 10.1016/j.joms.2015.02.027 25970513

[4] John B, Poorna TA, Joshna EK. Optimal depth of penetration to access the superior joint space in temporomandibular joint arthroscopy: a single institutional retrospective study. Journal of Maxillofacial and Oral Surgery. 2024; 23: 285–289. 10.1007/s12663-022-01815-1 PMC11001795 38601224

[5] González-García R. Categorizing temporomandibular joint arthroscopic procedures for a better clinical practice and teaching. Journal of Stomatology, Oral and Maxillofacial Surgery. 2024; 125: 101808. 10.1016/j.jormas.2024.101808 38423358

[6] Ward CKB, Hakim MA. Pearls and pitfalls of temporomandibular joint arthroscopy. In Amin D, Marwan H (eds.) Pearls and pitfalls in oral and maxillofacial surgery (pp. 275–282). Springer: Cham, Switzerland. 2024.

[7] Mc Cain JP, De La Rua H. A modification of the double puncture technique in temporomandibular joint arthroscopy. Journal of Oral and Maxillofacial Surgery. 1990; 48: 760–761. 10.1016/0278-2391(90)90069-e 2358958

[8] Murakami K, Hoshino K. Regional anatomical nomenclature and arthroscopic terminology in human temporomandibular joints. Okajima’s Japanese Journal of Anatomy. 1982; 58: 745–760. 10.2535/ofaj1936.58.4-6_745 7122015

[9] Del Castillo Pardo de Vera JL, Pampín Martínez M, Aragón Niño I, Navarro Cuéllar C, Cebrián Carretero JL. Navigation in surgical arthroscopy of the temporomandibular joint. British Journal of Oral and Maxillofacial Surgery. 2022; 60: 999–1001. 10.1016/j.bjoms.2022.03.005 35643567

[10] Zubiate Illarramendi I, Cabello A, Martínez-Sahuquillo Rico Á, Cariati P, Garcia Medina B. An exchange technique in temporomandibular joint (TMJ) arthroscopy to insert a larger operative cannula to facilitate advanced procedures. British Journal of Oral and Maxillofacial Surgery. 2024; 62: 97–100. 10.1016/j.bjoms.2023.10.011 37981521

[11] de Melo LA, Bezerra de Medeiros AK, Campos MFTP, Bastos Machado de Resende CM, Barbosa GAS, de Almeida EO. Manual therapy in the treatment of myofascial pain related to temporomandibular disorders: a systematic review. Journal of Oral & Facial Pain and Headache. 2020; 34: 141–148. 10.11607/ofph.2530 32255579

[12] Garrigós-Pedrón M, La Touche R, Navarro-Desentre P, Gracia-Naya M, Segura-Ortí E. Effects of a physical therapy protocol in patients with chronic migraine and temporomandibular disorders: a randomized, single-blinded, clinical trial. Journal of Oral & Facial Pain and Headache. 2018; 32: 137–150. 10.11607/ofph.1912 29694464

[13] Abboud WA. Novel guide device for temporomandibular joint arthroscopy. International Journal of Oral and Maxillofacial Surgery. 2020; 49: 1217–1219. 10.1016/j.ijom.2020.02.013 32171619

[14] Abboud W, inventor; Tel Hashomer Medical Research Infrastructure and Services Ltd. Ramat Gan (IL), assignee. Guide device suitable for performing temporomandibular joint arthroscopy. Israel: USPTO; US11517348B2. 29 November 2022.

[15] González LV, López JP, Díaz-Báez D, Martin-Granizo López R. Intraoperative complications in temporomandibular joint arthroscopy: A retrospective observational analysis of 899 arthroscopies. Journal of Cranio-Maxillo-Facial Surgery. 2022; 50: 651–656. 10.1016/j.jcms.2022.06.011 35842375

[16] Ângelo DF, Araújo RAD, Sanz D. Surgical complications related to temporomandibular joint arthroscopy: a prospective analysis of 39 single-portal versus 43 double-portal procedures. International Journal of Oral and Maxillofacial Surgery. 2021; 50: 1089–1094. 10.1016/j.ijom.2020.07.020 33495103

[17] González-García R, Monje F. Complications of temporomandibular joint arthroscopy. A critical appraisal of the literature. Journal of Cranio-Maxillo-Facial Surgery. 2024; 52: 1122–1132. 10.1016/j.jcms.2024.06.018 39030113

[18] Murakami KI. Current role of arthrocentesis, arthroscopy and open surgery for temporomandibular joint internal derangement with inflammatory/degenerative disease; -pitfalls and pearls-. Journal of Oral and Maxillofacial Surgery, Medicine, and Pathology. 2022; 34: 1–11.

[19] Murakami K, Ono T. Temporomandibular joint arthroscopy by inferolateral approach. International Journal of Oral and Maxillofacial Surgery. 1986; 15: 410–417. 10.1016/s0300-9785(86)80029-1 3091720

[20] Talaat WM, McGraw TA, Klitzman B. Relationship between the canthal-tragus distance and the puncture point in temporomandibular joint arthroscopy. International Journal of Oral and Maxillofacial Surgery. 2010; 39: 57–60. 10.1016/j.ijom.2009.11.016 20022729

[21] Caminiti MF, Driesman V, DeMontbrun S. The oral and maxillofacial objective structured assessment of technical skills (OMOSATS) examination: a pilot study. International Journal of Oral and Maxillofacial Surgery. 2021; 50: 277–284. 10.1016/j.ijom.2020.06.005 32694035

[22] Verde L, Muñoz-Guerra MF, Rodríguez-Campo FJ, Escorial V. Temporomandibular joint: approach to the intermediate space by triangulation with transillumination reference. Journal of Oral and Maxillofacial Surgery. 2023; 81: 684–688. 10.1016/j.joms.2023.02.008 36893793

[23] Danquah S, Choi D. Can remote learning and low fidelity simulation trainers improve confidence and fundamental skills in temporomandibular joint arthroscopy? International Journal of Oral and Maxillofacial Surgery. 2024; 52: 176–177.

[24] Chou J, Tenaglia M, Ho A, Valenti J, Davis C, Choi D. Can a low-fidelity arthroscopic simulator improve technical expertise in performing temporomandibular joint arthroscopy? Journal of Oral and Maxillofacial Surgery. 2024; 82: 1203–1211. 10.1016/j.joms.2024.06.180 39038595

[25] Monje Gil F, Hernandez Vila C, Moyano Cuevas JL, Lyra M, Pagador JB, Sanchez Margallo FM. Validation of a simulator for temporomandibular joint arthroscopy. International Journal of Oral and Maxillofacial Surgery. 2016; 45: 836–841. 10.1016/j.ijom.2016.01.010 26850940

[26] Peserico-DalFarra P, Gagliardi-Lugo AF. Training simulation for oral and maxillofacial surgeons based on the techniques of arthroscopy in the temporomandibular joint. British Journal of Oral and Maxillofacial Surgery. 2019; 57: 929–931. 10.1016/j.bjoms.2019.08.003 31445774

[27] Foo QC, Hariri F, Abdul Rahman ZA. Fabrication of a three-dimensional temporomandibular joint model for arthrocentesis and arthroscopy simulation. International Journal of Oral and Maxillofacial Surgery. 2021; 50: 1095–1099. 10.1016/j.ijom.2020.12.007 33422383

[28] Ângelo DF. Temporomandibular joint arthroscopy: inverted portal technique for more effective retrodiscal coblation. International Journal of Oral and Maxillofacial Surgery. 2022; 51: 1074–1077. 10.1016/j.ijom.2022.01.013 35101317

[29] Slavin AB. Comments on “single-cannula technique for operative arthroscopy using holmium:YAG laser”. Journal of Oral and Maxillofacial Surgery. 2012; 70: 1014. 10.1016/j.joms.2012.02.011 22538020

[30] Stringer DE, Park CM. Single-cannula technique for operative arthroscopy using holmium:YAG laser. Journal of Oral and Maxillofacial Surgery. 2012; 70: 49–50. 10.1016/j.joms.2011.07.021 21982691

[31] Martínez-Gimeno C, García-Hernández A, Martínez-Martínez R. Single portal arthroscopic temporomandibular joint discopexy: technique and results. Journal of Cranio-Maxillofacial Surgery. 2021; 49: 171–176. 10.1016/j.jcms.2021.01.004 33546966

[32] Liu Y, Wang P, Telha W, Jiang N, Bi R, Zhu S. Arthroscopic reduction and rigid fixation of the anteriorly displaced temporomandibular joint disc without reduction using titanium screw: a case series. Clinical Oral Investigations. 2024; 28: 156. 10.1007/s00784-024-05552-2 38376600

[33] Abboud WA, Givol N, Yahalom R. Arthroscopic lysis and lavage for internal derangement of the temporomandibular joint. Annals of Maxillofacial Surgery. 2015; 5: 158–162. 10.4103/2231-0746.175754 PMC4772553 26981463

[34] Badri O, Davis CM, Warburton G. Arthroscopic management and recent advancements in the treatment of temporomandibular joint disorders. British Journal of Oral and Maxillofacial Surgery. 2024; 62: 820–825. 10.1016/j.bjoms.2024.07.007 39181842

[35] Abboud W, Nadel S, Yarom N, Yahalom R. Arthroscopy of the temporomandibular joint for the treatment of chronic closed lock. The Israel Medical Association Journal. 2016; 18: 397–400. 28471560

